# Trade-off between reproduction and lifespan of the rotifer *Brachionus plicatilis* under different food conditions

**DOI:** 10.1038/s41598-017-15863-y

**Published:** 2017-11-13

**Authors:** Yunfei Sun, Xinying Hou, Xiaofeng Xue, Lu Zhang, Xuexia Zhu, Yuan Huang, Yafen Chen, Zhou Yang

**Affiliations:** 1 Jiangsu Key Laboratory for Biodiversity and Biotechnology, School of Biological Sciences, Nanjing Normal University, 1 Wenyuan Road, Nanjing, 210046 China; 2 State Key Laboratory of Lake and Environment, Nanjing Institute of Geography and Limnology, Chinese Academy of Sciences, 73 East Beijing Road, Nanjing, 210008 China

## Abstract

*Phaeocystis globosa*, one of the most typical red tide-forming species, is usually mixed in the food composition of rotifers. To explore how rotifers respond by adjusting life history strategy when feeding on different quality foods, we exposed the rotifer *Brachionus plicatilis* to cultures with 100% *Chlorella*, a mixture of 50% *P*. *globosa* and 50% *Chlorella*, or 100% *P*. *globosa*. Results showed that rotifers exposed to 100% *Chlorella* or to mixed diets produced more total offspring and had higher age-specific fecundity than those exposed to 100% *P*. *globosa*. Food combination significantly affected the net reproduction rates of rotifers. By contrast, rotifers that fed on 100% *P*. *globosa* or on mixed diets had a longer lifespan than those fed on 100% *Chlorella*. The overall performances (combining reproduction and lifespan together) of rotifers cultured in 100% *Chlorella* or mixed diets were significantly higher than those cultured in 100% *P*. *globosa*. In general, *Chlorella* favors rotifers reproduction at the cost of shorter lifespan, whereas *P*. *globosa* tends to extend the lifespan of rotifers with lower fecundity, indicating that trade-off exists between reproduction and lifespan under different food conditions. The present study also suggests that rotifers may have the potential to control harmful *P*. *globosa*.

## Introduction

Rotifer, as an important component of zooplankton community, plays an important role in linking the primary producers and higher trophic consumers^1^. Rotifers are considered as heterophagous microplanktonic filter feeder and can live in environments where resource types, quality, and abundance change dramatically. Under favorable food condition, rotifers generally reproduce rapidly^2^, that is, *Brachionus calyciflorus* had a maximal production rate when fed on *Chlorella vulgaris* at 750 µg mL^−1^ (wet weight). Facing unfavorable environments, rotifers can survive by adjusting their life history strategies. For example, *B*. *calycifloru*s would extend lifespan by reducing the growth and reproduction when feeding on bad food^3^. Kirk^4^ found the trade-off between reproduction and lifespan in the rotifers *Keratella cochlearis* and *Platyius quadricornus* under limited food. Based on the population abundance, reproduction and survival, rotifers are recently used for monitoring the toxicity of harmful algae, such as *Prorocentrum micans* and *Heterosigma akashiwo* ^5^. Some harmful algae at low concentrations could sustain the individual survival of rotifers, as well as the reproduction and population growth^5^.

Harmful algal blooms have become global environmental problems, which affect the balance of aquatic ecosystem and development in aquaculture and fishing industries^6,7^. The haptophyte *Phaeocystis globosa*, one of the most widespread marine algae, is a harmful bloom-forming phytoplankton^8^. It can grow well in the entire marine phytoplankton community from polar to temperate waters during the spring–summer transition^9^. *P*. *globosa* exists in two major morphotypes as follows: mucilaginous colonies and small single cells^10^. Like most *Chrysophyta* species, the small single cells have two flagella that help the cells move fast. The harmful effect of *P*. *globosa* derives from its ability to form red tide and produce hemolytic toxins^11,12^. Eight toxic compounds from *P*. *globosa* have been separated, some of which were lethal to aquatic animals, such as the brine shrimp *Artemia salina*, the juvenile *Epinephelus akaara* fish^13^, and the *Gadus morhua* larva^14^. *P*. *globosa* is generally not a good food for most zooplankton species^15^. However, during the early stage of red tide development, some of the zooplankton species, such as the copepods *Temora longicornis* and *Acartia clause*, could have high abundances^16^, indicating that copepods can survive well when fed with *P*. *globosa*. Some protozoa, such as ciliates could well graze the solitary cells of *P*. *globosa* ^17,18^.

To our knowledge, no report exists about the possible trade-off between reproduction and lifespan in the rotifers that fed on *P*. *globosa* to date. Considering that rotifer usually co-exists with *P*. *globosa* in nature and some zooplanktons survive on *P*. *globosa*, we hypothesized that (1) the rotifer *Brachionus plicatilis* can survive but reproduction may be affected when feeding on *P*. *globosa*, (2) trade-off exists between reproduction and lifespan of *B*. *plicatilis* under different food conditions, and (3) *B*. *plicatilis* may have the potential to control the harmful *P*. *globosa*. To test the hypotheses, we used both *P*. *globosa* (generally not a good food for zooplankton) and *Chlorella* sp. (generally a good food for rotifers) as experimental food to culture the widespread rotifer *B*. *plicatilis* under three different food combinations with the same dry weight, namely, 100% *Chlorella* sp. (100%C), a mixture of 50% *P*. *globosa* and 50% *Chlorella* sp. (50%C + 50%P), and 100% *P*. *globosa* (100%P), and recorded the life-history parameters. Results supported the hypotheses and indicated that *B*. *plicatilis* may have a potential to control the harmful *P*. *globosa*.

## Results

### Survival rate and lifespan

**Figure 1:**
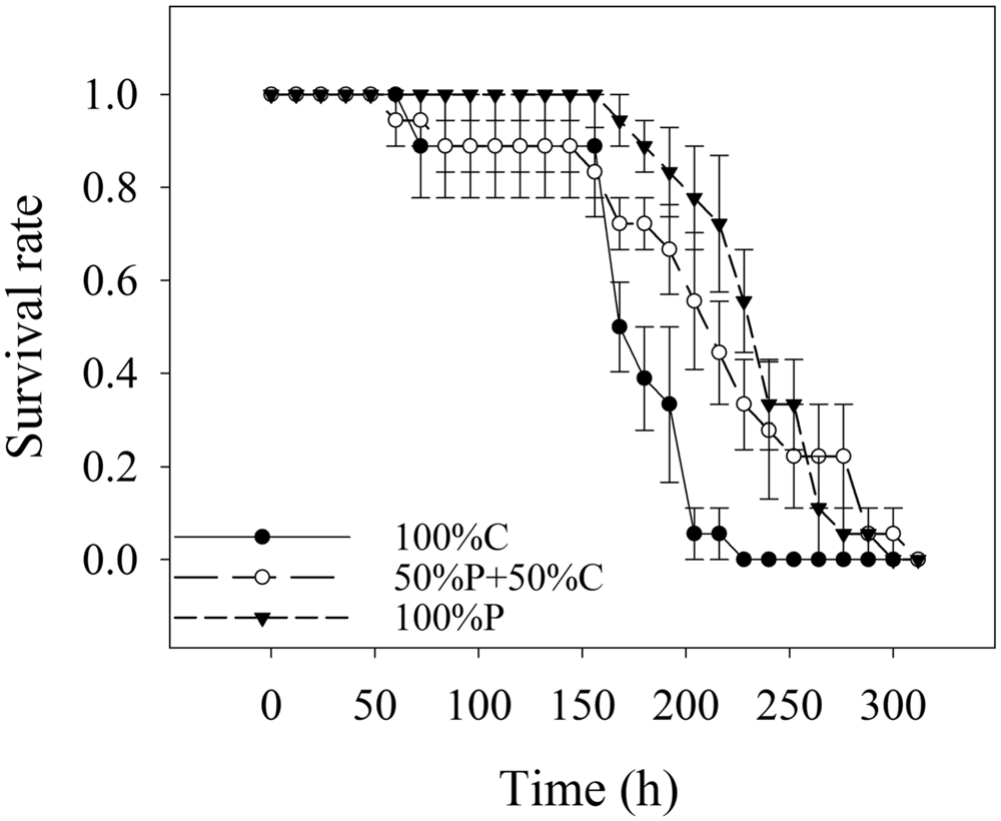
Survival rate of *B*. *plicatilis* at different culture times (12-h interval) with different food combinations (100% C, 50% C + 50% P, 100% P).

No significant difference was found in the three treatments before 150 h. After 150 h, the survival rate of rotifers in 100% C decreased sharply and was significantly lower than those in the other treatments (Fig. 1). However, no significant effect was observed between the rotifers fed with *P*. *globosa* and mixed food (*P* = 0.152).

**Figure 2:**
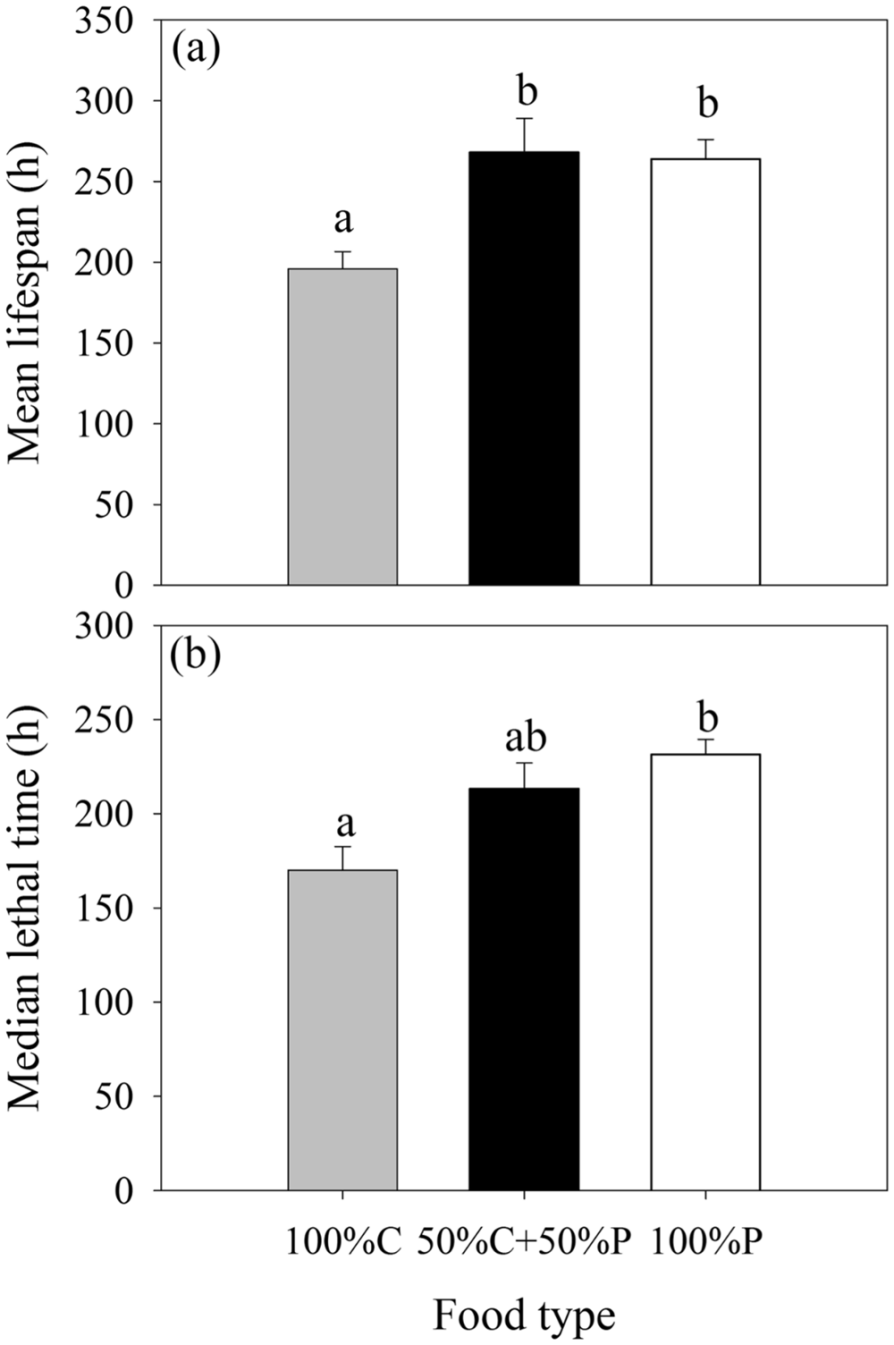
Mean lifespan (**a**) and the median lethal time (**b**) of *B*. *plicatilis* cultured with different food combinations (100% C, 50% C + 50% P, 100% P). Vertical lines represent standard error (*n* = 3). The significant differences are indicated by different lowercase letters (one-way ANOVA, *P* < 0.05).

The mean lifespan of rotifers cultured with 100% C (196 h) was significantly shorter than those in both 50% C + 50% P (*P* = 0.020) and 100% P (*P* = 0.016), but no significant difference was observed between lifespans in rotifers fed on 50% C + 50% P (268 h) and 100% P (264 h) (*P* = 0.860) (Fig. 2a). The median lethal time of rotifers cultured with 100% P was significantly longer than that of rotifers cultured with 100% C (*P* = 0.024). No significant difference in the median lethal time was observed between 50% C + 50% P and 100% P (*P* = 0.547) or between 100% C and 50% C + 50% P (*P* = 0.09) (Fig. 2b).

### Reproduction

**Figure 3:**
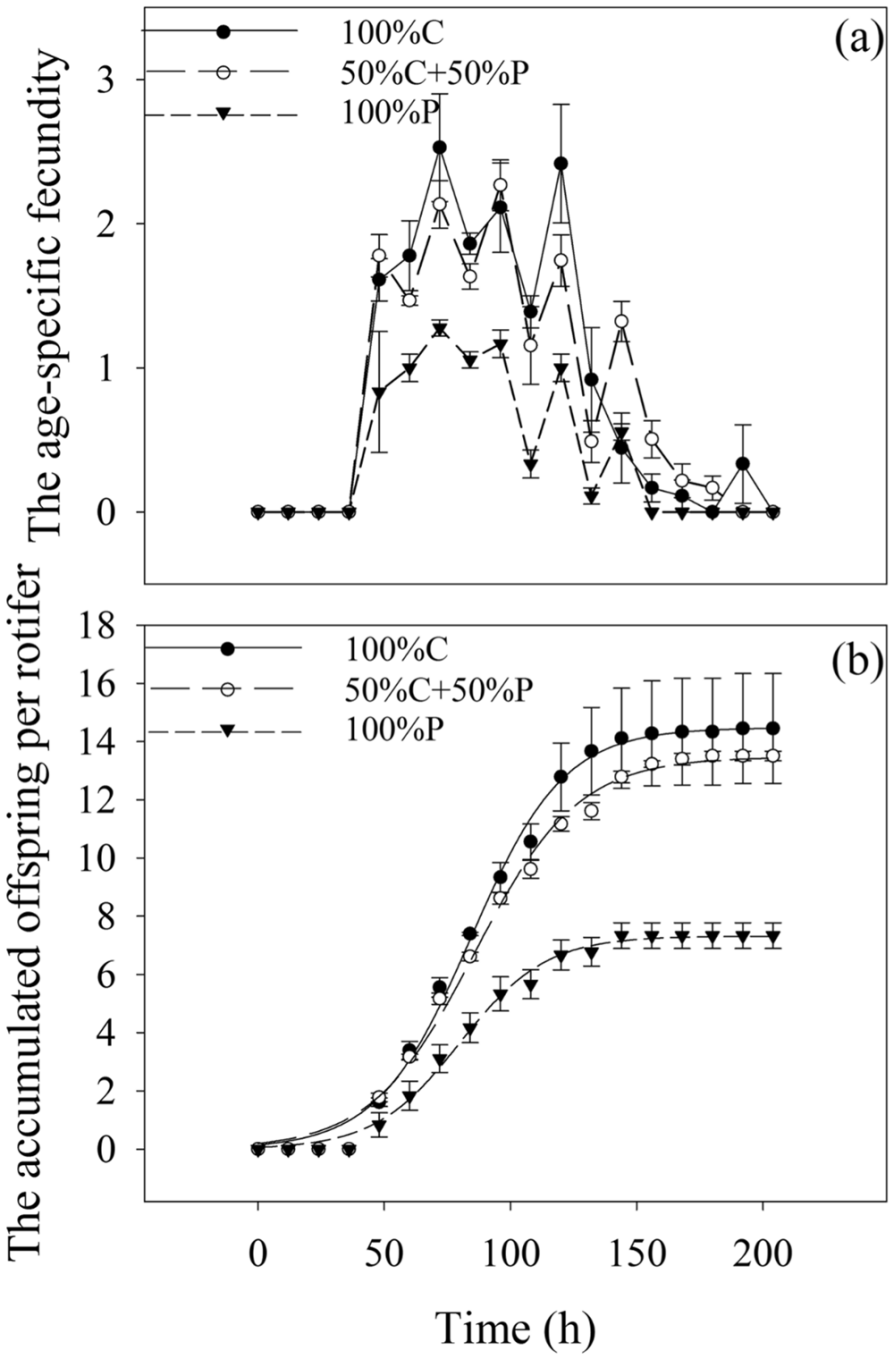
Age-specific fecundity (**a**) of *B*. *plicatilis* at different culture times (12-h interval) with different food combinations (100% C, 50% C + 50% P, 100% P) and the accumulated offspring per rotifer (**b**) of *B*. *plicatilis* cultured with different food combinations. Vertical lines represent standard error (*n* = 3). The significant differences are indicated by different lowercase letters (one-way ANOVA, *P* < 0.05).

The age-specific fecundity in all treatments increased with time, and then decreased after a plateau was reached (Fig. 3a). The accumulated total offspring of rotifers cultured with 100% P (maximum value: 7.33) significantly decreased by approximately 50% compared with those cultured in 100% C (maximum value: 14.44) and 50% C + 50% P (maximum value: 13.50) (*P* < 0.001), but no significant difference was observed between the rotifers cultured in 50% C + 50% P and 100% C (*P* = 0.091) (Fig. 3b).

The net reproduction rate (*R*_*0*_), generation time (*T*), and intrinsic rate of population increase (*r*_*m*_) are shown in Table 1. The *R*_*0*_ of rotifers in 100% P was lower than that of the rotifers in 50% C + 50% P (*P* = 0.016) or 100% C (*P* = 0.009), but no significant difference was observed in *R*_*0*_ of rotifers cultured with 50% C + 50% P and 100% C (*P* = 0.870). Similarly, the *r*_m_ of rotifers fed on 100% P was lower compared with that of the rotifers cultured with 50% C + 50% P (*P* = 0.019) or 100% C (*P* = 0.005), but no significant difference was observed between the rotifers in 50% C + 50% P and 100% C (*P* = 0.473). The generation time of the three food treatments had no significant difference (*P* = 0.622).

**Table 1:** Effects of food combination on the R_0_, T, and r_m_ of rotifers cultured with different food combinations. Significant differences are indicated by different lowercase letters.

Food combination	R_0_ (ind)	T (h)	r_m_ (h^−1^)
100% *Chlorella*	14.28 ± 1.81^a^	89.86 ± 5.66^a^	0.0294 ± 0.000403^a^
50% *Chlorella* + 50% *Phaeocystis*	13.5 ± 0.17^a^	93.66 ± 1.47^a^	0.0278 ± 0.0003997^a^
100% *Phaeocystis*	7.33 ± 0.44^b^	88.09 ± 3.62^a^	0.0227 ± 0.001506^b^

### Overall performance

**Figure 4:**
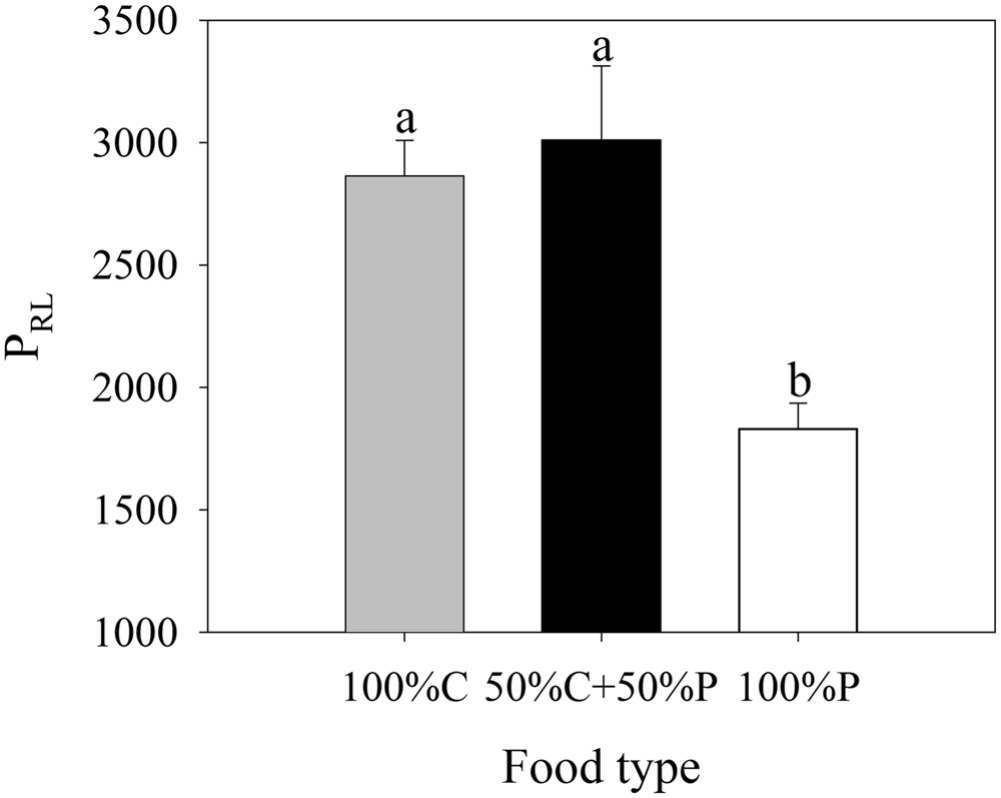
Overall performance (P_RL_) of *B*. *plicatilis* cultured with different food combinations (100% C, 50% C + 50% P, 100% P). Vertical lines represent standard error (*n* = 3). The significant differences are indicated by different lowercase letters (one-way ANOVA, *P* < 0.05).

As for the overall performance, the rotifers cultured with 100% P was significantly lower than those in other two treatments (*P* < 0.001), but no significant difference was observed between the rotifers fed on 50% C + 50% P and 100% C (*P* = 0.082) (Fig. 4).

## Discussion

The main results of the current study showed that *Chlorella* sp. favored rotifer reproduction with shorter lifespan, whereas *P*. *globosa* tended to extend lifespan with lower reproduction, which demonstrated that trade-off exists between the reproduction and lifespan of the rotifer *B*. *plicatilis* under different food conditions. However, the overall performance of the rotifers completely cultured with *P*. *globosa* was significantly lower than rotifers fed on *Chlorella* sp. or mixed foods.

Changes in reproduction and lifespan of rotifer under different foods in our experiments were comparable to the study by Yoshinaga *et al*.^3^, which suggested that the rotifer *B*. *plicatilis* would have a longer lifespan with the simultaneously decreased reproduction rate when they were badly fed or starved. Energy allocation models provide a very useful framework to investigate the changes in rotifer life history^19,20^. As all energy used to new growth and reproductive effort come from the ingested food, rotifers should achieve a two-way trade-off when nutrition is limited^21^, in which the lifespan and reproduction were clearly negatively related^22^. Our result indicated that when the reproduction rate was high, the mean lifespan was short, confirming the trade-off between reproduction and lifespan when rotifers were fed on different types of food, even under sufficient food conditions. As this trade-off generally occurs when rotifers confront stressful environments^23^, *P*. *globosa* was not as good food as the *Chlorella* to *B*. *plicatilis*.

As food for zooplankton, the quality of algae depends on the nutrition, toxicity, and morph^24^. In the present study, 100% *Chlorella* or mixed alga caused the rotifers to produce more offspring. Conversely, 100% *P*. *globosa* caused the rotifers to have a lower reproduction rate. Algal size influences the filtration and ingestion rate of zooplankton. Rotifers feeding with large algae generally had a better reproduction than that feeding with small algae^25^. However, in the present study, *P*. *globosa* is larger than the *Chlorella* sp. (7.2 µm vs. 4.8 µm in diameter), indicating other characters of *P*. *globosa* rather than the algal size affecting the rotifers. *P*. *globosa* lacks unsaturated fatty acid, which is necessary for the growth and reproduction of rotifers^26–28^. In addition, *Phaeocystis* sp. were found to be toxic toward *Artemia salina*, *Epinephelus akaara* fish^13^, and *Gadus morhua* larvae^14^. Thus, the poor quality and toxic property of *P*. *globosa* may have contributed to the inhibited reproduction of rotifers^5,29^.

When the rotifers were fed on 50% *Chlorella* + 50% *P*. *globosa*, they had longer lifespan compared with those fed on 100% *Chlorella* and higher accumulated offspring compared with those fed on 100% *P*. *globosa*. The overall performance of rotifers under 50% *Chlorella* + 50% *P*. *globosa* was nearly similar to those feeding on 100% *Chlorella*, indicating that the energy that these rotifers obtained is comparable under the two food conditions. The rotifers under this food combination may partly obtain both the advantages from the two species of algae. For example, some algal extracts and antioxidants in *P*. *globosa* could extend the lifespan of rotifers^30,31^. From the above results, it was concluded that rotifers may have excellent potential to control *P*. *globosa*, especially under mixed algal community.

In summary, food combination significantly affected the reproduction and lifespan of rotifers. *Chlorella* favors rotifers reproduction at the cost of shorter lifespan, whereas *P*. *globosa* tends to extend the lifespan of rotifers with lower fecundity, indicating that trade-off exists between reproduction and lifespan under different food conditions. This study also suggests that rotifers may have the potential to control the harmful *P*. *globosa*.

## Materials and Methods

### Plankton and cultivation

Both *P*. *globosa* and *Chlorella sp*. were obtained from Xiamen University and were cultivated in autoclaved seawater (NaCl 24.54 g, KBr 0.1 g, KCl 0.7 g, H_3_BO_3_ 0.003 g, Na_2_SO_4_ 4.09 g, NaHCO_3_ 0.185 g, NaF 0.003 g, CaCl_2_∙2H_2_O 1.54 g, MgCl_2_∙6H_2_O 11.10 g, and SrCl_2_∙6H_2_O 0.017 g in every 1 L deionized water; salinity 33‰; pH = 8.3) with f/2 medium at 25 °C under a 14 h:10 h light:dark cycle at 50 µmol photons m^−2^s^−1^. The salinity of seawater The resting eggs of *B*. *plicatilis* were obtained from the Chinese Academy of Sciences and hatched in beakers with the autoclaved seawater (salinity 33‰, DO > 6.0 mg/L) at 25 °C in a 14 h:10 h light:dark cycle at 20 µmol photons m^−2^ s^−1^. Before the experiment, *B*. *plicatilis* were fed on *Chlorella* sp. (3.0 × 10^6^ cells mL^−1^) under similar conditions as described above.

### Experimental design

Three treatments were arranged, and each treatment contained six neonates (<8 h old). The three different food treatments comprised 100% C (3.0 × 10^6^ cells mL^−1^), 50% C + 50% P (1.5 × 10^6^ cells mL^−1^ + 2.75 × 10^6^ cells mL^−1^), or 100% P (5.5 × 10^6^ cells mL^−1^) with a same dry weight (0.22 mg mL^−1^). Each neonate in every treatment was raised in 1 mL of autoclaved seawater. The experiment was conducted in 24-welled culture plates. Every 12 h, the living rotifers and the new-born neonates were counted, and the dead maternal individuals and new-born neonates were removed. Every 24 h, the maternal rotifers were transferred into fresh seawater with the corresponding foods. The experiments were run in triplicates and under similar environmental conditions as described above. The experiments proceeded until all individuals of each cohort died. All 24-welled culture plates were shaken every 12 h to avoid the sedimentation of algae. We calculated the net reproduction rate (*R*_*0*_), generation time (*T*), intrinsic rate of population increase (*r*_*m*_), and mean lifespan (*L*) according to Krebs^32^. To assess the total energy output of rotifers that were cultured in different food combinations, we adopted an index, overall performance (P_RL_), combining reproduction and lifespan together, based on the fitness formula. The demography parameters were calculated using the formula:

1 $$L=\sum {l}_{x}$$

2 $${R}_{{0}}=\sum {l}_{x}{m}_{x}$$

3 $$T=\sum x{l}_{x}{m}_{x}/{R}_{{0}}$$

4 $${r}_{m}=\,\mathrm{ln}\,{R}_{{0}}/T$$

5 $${P}_{RL}=N\,L$$

where *x* is the culture time, *l*_*x*_ is age-specific survival, *m*_*x*_ is the age-specific fecundity, and *N* is the total offspring per rotifer.

### Statistical analyses

Data were presented as the mean values ± standard error. The effect of food combination on mean lifespan, the accumulated offspring per rotifer, the median lethal time, *R*_*0*_, *t, R*_*m*_, and P_RL_ were analyzed by one-way ANOVA. All statistical analyses were performed in Sigmaplot 11.0.
